# The role of exosomes and exosomal microRNA in diabetic cardiomyopathy

**DOI:** 10.3389/fendo.2023.1327495

**Published:** 2024-01-12

**Authors:** Shiyu Gao, Yue Dong, Chentao Yan, Tianhao Yu, Hongbo Cao

**Affiliations:** College of Traditional Chinese Medicine, Tianjin University of Traditional Chinese Medicine, Tianjin, China

**Keywords:** microRNA, exosome, diabetic cardiomyopathy, angiogenesis, cardiac fibrosis

## Abstract

Diabetic cardiomyopathy, a formidable cardiovascular complication linked to diabetes, is witnessing a relentless surge in its incidence. Despite extensive research efforts, the primary pathogenic mechanisms underlying this condition remain elusive. Consequently, a critical research imperative lies in identifying a sensitive and dependable marker for early diagnosis and treatment, thereby mitigating the onset and progression of diabetic cardiomyopathy (DCM). Exosomes (EXOs), minute vesicles enclosed within bilayer lipid membranes, have emerged as a fascinating frontier in this quest, capable of transporting a diverse cargo that mirrors the physiological and pathological states of their parent cells. These exosomes play an active role in the intercellular communication network of the cardiovascular system. Within the realm of exosomes, MicroRNA (miRNA) stands as a pivotal molecular player, revealing its profound influence on the progression of DCM. This comprehensive review aims to offer an introductory exploration of exosome structure and function, followed by a detailed examination of the intricate role played by exosome-associated miRNA in diabetic cardiomyopathy. Our ultimate objective is to bolster our comprehension of DCM diagnosis and treatment strategies, thereby facilitating timely intervention and improved outcomes.

## 1 Introduction

Diabetes (diabetes mellitus, DM) is a metabolic disease characterized by chronic hyperglycemia resulting from pancreatic β-cell dysfunction and peripheral insulin resistance caused by genetic and environmental factors. This leads to disturbances in glucose metabolism and the development of chronic low-grade inflammation (1). Diabetes represents the third major threat to human health, following cardiovascular disease and cancer. According to the International Diabetes Federation (IDF), the global diabetic population has reached 537 million, with approximately 6.7 million people succumbing to diabetes or its complications annually (2). Being a prevalent ailment with a substantial patient population and protracted course, diabetes poses significant harm, profoundly impacting the well-being and health security of all mankind, thereby ranking as one of the world’s most pressing public health challenges. In China, the number of DM patients is approximately 116.4 million, securing its place as the highest worldwide. This surge in patient numbers not only severely affects the populace’s health but also imposes a substantial economic burden on patients, their families, and society at large (3). Diabetes is commonly classified into four main categories: type I diabetes, type II diabetes, gestational diabetes, and other specific types. Prolonged hyperglycemia and enduring metabolic disorders give rise to a cascade of complications. A multitude of studies has consistently revealed a close association between hyperglycemia and microangiopathy across all diabetes types. Diabetes can lead to cardiac autonomic dysfunction and myocardial hypertrophy, resulting in the development of diabetic cardiomyopathy (4, 5). According to diabetes statistics, the mortality rate resulting from cardiovascular complications in individuals with diabetes mellitus remains alarmingly high. Approximately 44% of patients diagnosed with type 1 diabetes mellitus (T1DM) succumb to cardiovascular complications, and this percentage climbs even higher in the case of T2DM, reaching a staggering 52% of total fatalities (6). Research findings have illuminated the pathogenesis of diabetic cardiomyopathy (DCM), which commences with structural damage and abnormalities in cardiomyocytes. This leads to cardiomyocyte hypertrophy, alterations in ventricular wall thickness and tension, the transition of the heart from diastolic dysfunction to systolic dysfunction, myocardial diffuse fibrosis, and ultimately culminates in end-stage heart failure (7).

Exosomes, a subgroup of extracellular vesicles, function as conveyors for transporting biomolecules and facilitating intercellular communication. They exert a profound influence on the onset and progression of a diverse array of diseases. Recent studies have unveiled the pivotal role that exosomes play in DCM, with microRNA (miRNA) being recognized as the principal signaling molecule enabling exosomes to enact their effects. Upon loading into exosomes, miRNA exerts its influence on the biological function of recipient cells. Presently, exosomal miRNA is acknowledged to wield a critical role in cardiovascular diseases, cancer, and neurodegenerative diseases. Consequently, increasing attention is being devoted to the prevention and treatment of DCM. This article comprehensively reviews the most recent advancements in research concerning the role of exosome miRNA in DCM, aiming to stimulate novel ideas for DCM treatment and further research endeavors (8).

## 2 Exocrine and miRNA

### 2.1 Composition and function of exosomes

**Figure 1:**
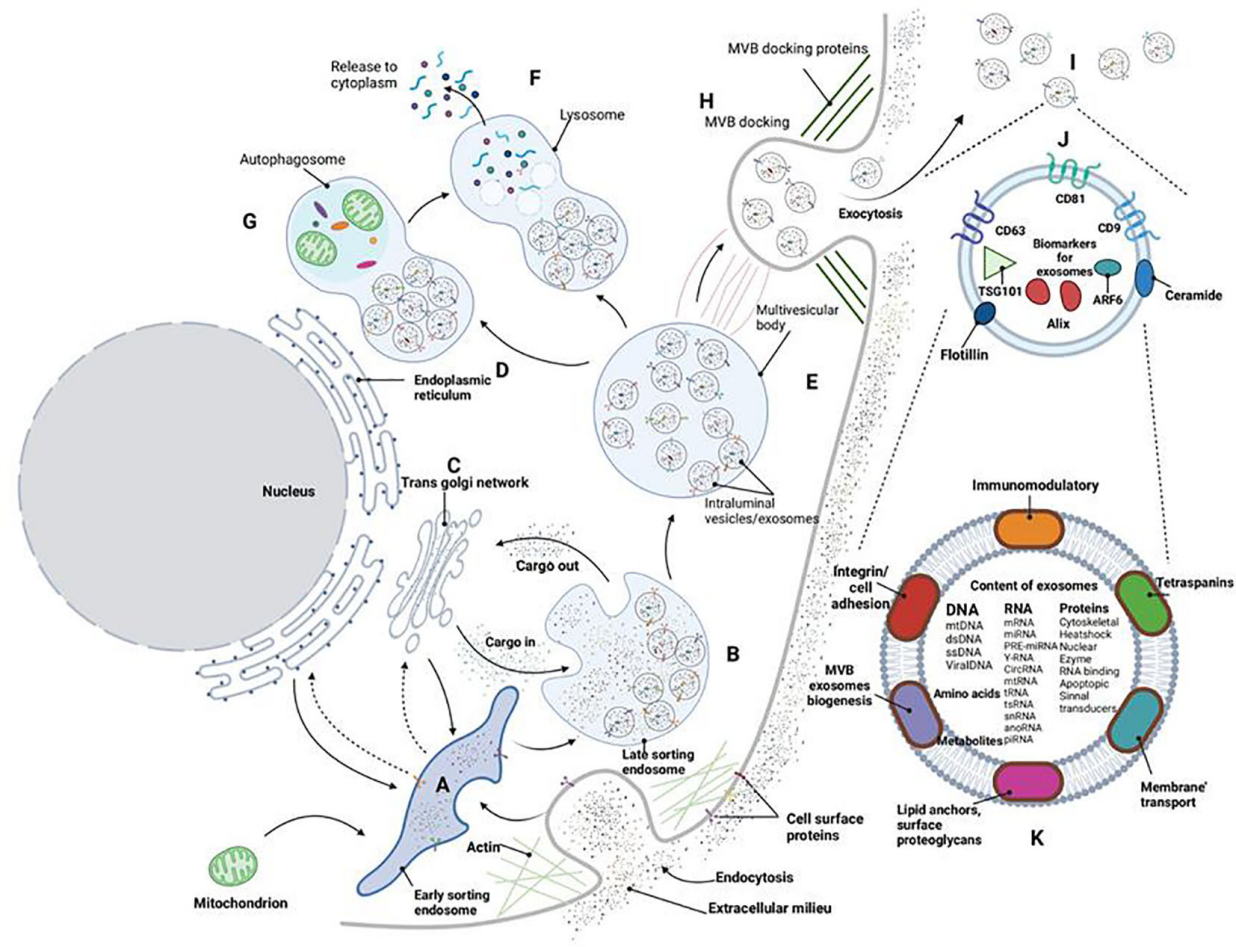
Exosome biogenesis. Cell membrane invagination initiates the formation of early inner nuclear bodies **(A)**. Various buds enter the endosomal lumen, commencing the generation of exosomes/intracavitary vesicles **(B)** within late sorting endosomes. Cargo molecules are translocated between the trans-Golgi network **(C)**, endoplasmic reticulum **(D)**, and the late sorting endosome **(B)**. Collectively, these processes culminate in the creation of multivesicular bodies **(E)** that contain fully developed luminal vesicles/exosomes. Multivesicular bodies can subsequently undergo intracellular processing via lysosomes **(F)** or autophagosomes **(G)**, resulting in the breakdown of the multivesicular body’s components within the cell. Alternatively, these polyvesicles can dock **(H)** and fuse with the cell membrane, releasing exosomes **(I)** into the extracellular space. Exosomes consistently express various surface markers **(J)** that can be used for characterization. The functions of exosomes **(K)** are contingent on the diverse cargoes they encapsulate within the exobody.

The exocrine body is an extracellular vesicle with a lipid bilayer, ranging in diameter from 40 to 160nm, and originates from endosomes. It is primarily composed of nucleic acids and proteins and can be found in blood, urine, cerebrospinal fluid, and other body fluids (9). Exocrine bodies serve as signal vesicles in autocrine, endocrine, paracrine, or distal communication modes, carrying various molecules, including lipids, mRNA, small RNAs (such as miRNA and miR), membrane-binding proteins, and other regulatory RNAs. They play a role in cell-to-cell signal transmission and immune responses (10, 11). Exosomes enter the cell through three different pathways: they either enter the recipient cell and release their “cargo” (such as small molecule drugs, proteins, nucleic acids, etc.) into the cytoplasm, forming polyvesicular bodies; release their “cargo” into the cytoplasm and fuse with the cytoplasmic membrane; or their ligands bind to specific receptors on the cell membrane, carrying the “cargo” into the cell through signal transduction pathways and endocytosis pathways (12). The release and content of exosomes are influenced by various physiological factors and cellular conditions, including oxidative stress, hypoxia, and intracellular calcium levels. With ongoing research, exosomes have gained significant attention in the fields of stem cells, immunity, targeted drug delivery, and more. Researchers have discovered associations between exosomes and their contents with inflammatory responses, immune surveillance, carcinogenesis, and metabolic diseases. It is anticipated that exosomes could become a new non-invasive marker for disease diagnosis and offer a novel and feasible approach to disease treatment from an etiological perspective. Additionally, considering the excellent biocompatibility and low toxicity of exosomes, they are expected to become ideal nano-carriers for drugs (as summarized in **Figure 1**).

Exosomes play a crucial role in the pathogenesis and potential treatment of DCM. Various types of exosomes can promote a range of physiological and pathological processes, including embryonic development, vascular development, inflammatory response, ischemia-reperfusion injury, apoptosis, and cardiac remodeling/fibrosis (13, 14). Exosomes released by diabetic cardiomyocytes reduce angiogenesis in diabetic myocardium by transferring harmful factors. These factors can induce, amplify, and transmit downstream anti-angiogenic effects to cardiac endothelial cells, leading to impaired angiogenesis and promoting the development of DCM (15). Exosomes can also have a therapeutic impact in DCM. Some studies have revealed that exosomes activate the phosphorylation of ERK1/2, P38MAPK, and Hsp27, directly protecting primitive cardiomyocytes from insulin resistance through the interaction between Hsp70 and the cytoplasmic membrane TLR4 (16).

### 2.2 Exocrine miRNA

MiRNA is a short-stranded, endogenous, non-coding RNA composed of approximately 22 nucleotides. After binding to the 3’ untranslated region (UTR) of mRNA, it reduces the expression of target gene proteins and regulates the phenotype and function of recipient cells by promoting mRNA degradation or inhibiting translation. Exosomal miRNA is protected from RNase degradation by a phospholipid bilayer, which is advantageous for the isolation, extraction, and storage of exosomal miRNA. As exosomes circulate, the miRNA contained within them can be absorbed by neighboring or distant cells, regulating the recipient cells to fulfill specific roles. The transfer of intercellular information through circulating vesicles is regarded as the third pathway of signal transduction, and it plays a crucial regulatory role in cell proliferation, differentiation, migration, as well as disease initiation and progression (17).

In cardiovascular diseases, there is a wide range of miRNA expressions in cardiomyocytes. Abnormally expressed miRNA can regulate gene expression through the degradation or inhibition of mRNA post-transcriptional translation, and they participate in various pathophysiological mechanisms, including cell proliferation and differentiation, apoptosis, cytokine synthesis, and metastatic paracrine signaling (18). These miRNAs have different regulatory effects on various molecular pathways in different cardiovascular diseases. Some of them play a positive, protective role by either promoting their expression or inhibiting their expression. Studies have indicated that in the context of diabetes, exosomal miRNA is absorbed by recipient cells, taking part in the regulation of pancreatic β-cell injury and insulin resistance, thereby exerting its biological function (19). This ultimately influences the occurrence and development of diabetes-related complications such as DCM.

## 3 Exocrine miRNA and diabetic cardiomyopathy

Diabetic cardiomyopathy is a unique form of cardiomyopathy characterized by myocardial fibrosis, cardiomyocyte hypertrophy, and apoptosis. The early manifestation of DCM is cardiac diastolic dysfunction, which is associated with ventricular hypertrophy, high end-diastolic volume and pressure, as well as cardiac extracellular matrix remodeling. Collagen and advanced glycation end products (AGEs) interact in diabetes, leading to changes in myocardial hypertrophy and ventricular diastolic disorders. Additionally, persistent hyperglycemia disrupts mitochondrial energy metabolism, exacerbating myocardial hypertrophy, cardiomyocyte apoptosis, and fibrosis. When myocardial hypertrophy becomes too extensive to compensate for apoptosis and fibrosis, systolic dysfunction ensues (20). Insulin resistance, hyperinsulinemia, and hyperglycemia are independent risk factors for DCM (21).

**Figure 2:**
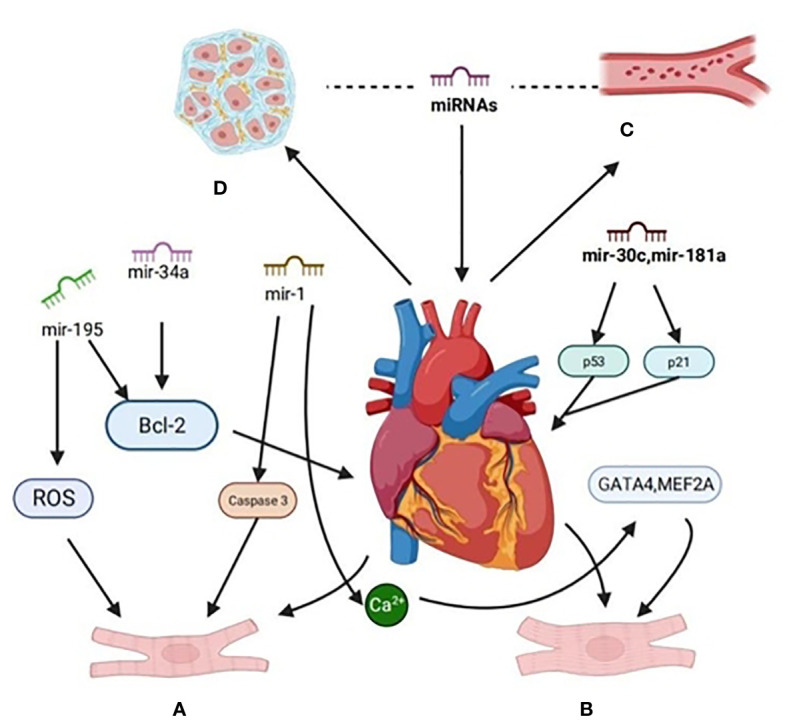
Signaling pathways are regulated by miRNA in diabetic cardiomyopathy. **(A)** The expression of miR-34a in H9c2 of rat cardiomyocytes treated with high glucose was up-regulated, the expression of miR-34a-targeted anti-apoptosis protein (Bcl-2) decreased, and the apoptosis rate of H9c2 cells increased. Overexpression of miR-195 can down-regulate the expression of Bcl-2,miR-195, reduce the production of ROS and inhibit apoptosis. miR-1 was up-regulated, and the activity of pro-apoptotic factor caspase-3 was increased. **(B)** MiR-30c and miR-181a cooperatively regulate p53-p21 pathway, improve cardiac structure and function, and pathological hypertrophy in diabetes-induced cardiac hypertrophy.MiR-1 delays the process of cardiomyocyte hypertrophy by negatively regulating calcium signal components calmodulin, cardiac transcriptional regulatory factor (GATA4) and cardiomyocyte enhancer factor 2A (MEF2A). **(C)** MiRNA inhibits angiogenesis by inhibiting the proliferation and migration of vascular endothelial cells,it can also promote angiogenesis. **(D)** Some miRNA can inhibit myocardial fibrosis through related pathways and proteases.

Research has revealed a variety of miRNA expressions in cardiomyocytes. Aberrantly expressed miRNAs can regulate gene expression through the degradation or inhibition of post-transcriptional mRNA translation, affecting cardiac function (22, 23). These miRNAs exert different regulatory effects on various molecular pathways in different cardiovascular diseases, such as myocardial infarction, DCM, arrhythmia, atherosclerosis, and more. Some of them play a positive, protective role by either promoting their expression or inhibiting their expression. Recent research indicates that in numerous diabetic animal models, overexpression of downregulated miRNAs or inhibition of upregulated miRNAs can alleviate the progression of DCM, albeit with certain limitations (24). Thus, identifying the pathogenesis and effective regulatory targets of DCM is of paramount importance. As a result, this paper discusses the potential mechanisms and effective regulatory targets involving exosomal miRNAs in the context of diabetic cardiomyopathy (as summarized in **Figure 2**).

### 3.1 Exocrine miRNA and cardiomyocyte apoptosis

Cardiomyocytes maintain homeostasis by controlling protein synthesis, substrate utilization, and cell survival. This balance is disrupted in DCM, leading to an increased rate of cardiomyocyte apoptosis. Multiple miRNAs regulate cardiomyocyte apoptosis. Furthermore, exosomal miRNAs mediate intercellular communication and can influence various cellular processes, including cell survival, as well as repair and regeneration of damaged myocardial tissue.

It has been observed that miR-30c and miR-181a are underexpressed in diabetic patients, DCM rat models, and cardiomyocytes treated with high glucose. p53 is an effective target of miR-30c and miR-181a, and the reduction in miRNA levels is closely linked to the activation of the p53 pathway, resulting in myocardial hypertrophy and apoptosis (25). Similarly, the upregulation of miRNA also plays a significant role in apoptosis induced by cardiac mitochondria. Studies have confirmed that miR-30d can directly target the expression of forkhead transcription factor 03a, leading to a decrease in the expression of downstream apoptosis inhibitors and apoptosis proteases, accompanied by an increase in the expression of cysteine proteases (26, 27).

Additionally, Zhu discovered that miR-34a expression was upregulated in H9c2 cells, leading to decreased expression of its target anti-apoptotic protein, Bcl-2, and an increased apoptosis rate in rats treated with high glucose (28). Furthermore, by injecting miR-195 mimic and miR-195 inhibitors into diabetic mice, they found that overexpression of miR-195 could target the downregulation of Bcl-2, while downregulation of miR-195 reduced the production of ROS and inhibited cell apoptosis. In the hearts of STZ-induced diabetic mice, miR-1 was upregulated, leading to downregulation of its target proto-oncogene serine/threonine protein kinase and Bcl-2, and an increase in the activity of the proapoptotic factor caspase-3 (29).

The researchers inhibited the release of exosomes from cardiac myocytes *in vivo* using ceramidase inhibitors. They discovered that exosomes released by diabetic cardiomyocytes could carry disease signals, and the signals received by neighboring endothelial cells led to impaired angiogenesis, ultimately promoting the development of DCM. Furthermore, the injection of adipocyte-derived EXsmiR-130b-3p into the myocardium of diabetic mice with myocardial ischemia-reperfusion injury significantly increased cardiomyocyte apoptosis and expanded the area of myocardial infarction. This is due to miR-130b-3p’s negative regulation of the expression of 5-adenine nucleotide-dependent protein kinase α2, which inhibits the expression of several anti-apoptotic and cardioprotective molecules in cardiomyocytes, exacerbating diabetic myocardial ischemia-reperfusion injury (30).

### 3.2 Exocrine miRNA and changes of myocardial hypertrophy

Previous studies have demonstrated that several cardiovascular diseases, including DCM, can lead to cardiomyopathic hypertrophy. In recent years, there has been extensive research on the role of miRNAs in cardiomyopathic hypertrophy. Some researchers have identified pathological changes in the miRNA expression profiles in the hearts of hypertrophic mice, indicating that various miRNAs can inhibit the occurrence and development of myocardial hypertrophy in DCM (31). Zhang discovered that miR-30c and miR-181a cooperate in regulating the p53-p21 pathway in diabetes-induced cardiac hypertrophy (32). Overexpression of miR-30d improved the hypertrophy of cardiomyocytes induced by PE and AngII in mice and neonatal rats, leading to improvements in heart structure and function, along with decreased levels of miR-30d in the serum of patients with chronic heart failure. Overexpression of miR-30d significantly ameliorated the pathological hypertrophy of cardiomyocytes derived from human embryonic stem cells (33). Feng believes that the level of miR-133a in hypertrophic myocardium is downregulated under high glucose conditions, while overexpression of miR-133a can mitigate the hypertrophic changes in cardiomyocytes (34). Additionally, Ma et al. demonstrated that miR-1 delayed the process of cardiomyocyte hypertrophy by negatively regulating calcium signal components, including calmodulin, cardiac transcriptional regulatory factor (GATA4), and cardiomyocyte enhancer factor 2A (MEF2A) (35). MiR-150 inhibits cardiomyocyte hypertrophy induced by high glucose by targeting the transcriptional coactivator p300 (36). Conversely, some miRNAs exhibit negative effects on myocardial health, such as miR-208a, which can upregulate β-myosin heavy chain by inhibiting the expression of myostatin and GATA4, thereby worsening myocardial hypertrophy (37). MiR-133 is the first miRNA to be identified in studies of diabetic cardiac complications. MiR-133 controls cardiomyocyte hypertrophy by acting on different target genes, including Cdc42 (a signal transduction kinase), Nelf-A/WHSC2 (a nuclear factor related to heart development), and RhoA (a GDP/GTP exchange protein that regulates cardiac hypertrophy) (38). The miR-1/mitochondrial calcium axis may be involved in the dynamic adaptation of cardiomyocytes to hypertrophy. MiRNAs play multiple roles in the regulation of myocardial hypertrophy, offering a potential new approach for early diagnosis, prevention, and treatment of DCM in the future.

### 3.3 Exocrine miRNA and vascular endothelial injury

The initial step in the development of diabetic vascular complications is vascular endothelial dysfunction, which is primarily characterized by inflammation (39). The Dicer protein is a key enzyme in miRNA maturation. Knocking out Dicer results in the loss of miRNAs in endothelial cells, which in turn affects neovascularization in embryos. MiRNAs are vital for maintaining the function of endothelial cells. Notable miRNAs in this context include miR-21, miR-24, miR-34a, miR-92a, miR-126, miR-200b, and miR-210 (40). This group of miRNAs inhibits angiogenesis by reducing the proliferation and migration of vascular endothelial cells. For instance, miR-21 inhibits vascular endothelial cell proliferation by targeting PPAR-α and RhoB (41). MiR-200b hampers the proliferation of vascular endothelial cells by downregulating the expression of Ets-1, VEGF, VEGFR-2, and GATA2 genes. Additionally, vascular smooth muscle cells (VSMCs) represent essential components of the vascular wall, primarily located in the intima of the vascular wall, and they play a role in blood pressure regulation through contraction. An increasing body of evidence suggests that phenotypic transformations occur in VSMCs in the context of diabetes, leading to diabetic vascular complications and further abnormalities in vascular structure and function. As a result, the phenotypic transformation of VSMCs is a critical research focus for the prevention and treatment of diabetic vascular complications.

A study established a rat model of type 2 diabetes through a high-glucose and high-fat diet combined with streptozotocin injection (42). After an 8-week aerobic exercise regimen for rats, it was observed that the transformation of vascular smooth muscle cells (VSMCs) into a synthetic phenotype was reversed. Concurrently, the expression of miR-181b was inhibited, while the expression of PTEN was up-regulated. Diabetes stimulates the expression of vascular PI3K protein. Aerobic exercise not only up-regulates the expression of vascular PTEN but also down-regulates the expression of vascular AKT. This suggests that aerobic exercise inhibits the downstream AKT signal by activating the miR-181b/PTEN pathway, thereby mitigating vascular injury. The potential mechanism behind this lies in exercise’s regulation of the miR-181b/PTEN pathway.

Furthermore, CircRNA WDR77 can regulate the proliferation of vascular smooth muscle under high-glucose conditions and subsequently affect miRNA-124 and fibroblast growth factor 2 (FGF2), forming the circWDR77-miRNA-124-FGF2 regulatory pathway (43). Exosomes from diabetic cardiomyocytes are transferred to endothelial cells, resulting in the up-regulation of miR-320 and the down-regulation of E26 transcription factor 2 (IGF)-1, heat shock protein (HSP) 20, and insulin-like growth factor (Ets2). Therefore, these findings suggest that exosomes isolated from diabetic cardiomyocytes reduce angiogenesis in the diabetic myocardium by transferring harmful factors. These harmful factors can induce, amplify, and transmit downstream cascades of anti-angiogenic effects to cardiac endothelial cells.

### 3.4 Exocrine miRNA and neovascularization

Neovascularization is a crucial process for reconstructing the collateral circulation in ischemic myocardium. MiR-146a plays a role in regulating the inflammatory signal pathway of TLR/IL1R by acting on the downstream target gene interleukin-1 receptor-associated kinase 1 (IRAK1). Reduced expression of miR-146a further inhibits angiogenesis, diminishes glucose uptake by cardiomyocytes, affects myocardial blood supply and energy metabolism, ultimately leading to ventricular remodeling and heart failure (44). MiR-126 and miR-21, as angiogenic factors, are down-regulated in patients with chronic heart failure, resulting in endothelial cell dysfunction. Elevating their expression through consistent aerobic exercise can promote angiogenesis (45). Exosomes rich in miR-294 can significantly improve myocardial necrosis in ischemic cardiomyopathy, increase new microangiogenesis, stimulate myocyte proliferation, and enhance myocardial survival after myocardial infarction. This effect lasts for 6-8 weeks, indicating a longer half-life of exosomes (46). Cardiomyocyte-enriched exosomes containing miR-320 exhibit anti-angiogenic properties. Research has shown that exosomal miR-320 released by central muscle cells of T2DM mice can be transferred to mouse cardiac endothelial cells, impairing the function of adjacent cardiac endothelial cells by down-regulating the expression of insulin-like growth factor 1 (IGF-1), heat shock protein 20 (HSP-20), and E26 transcription factor 2 (E-twenty-six 2). This also hampers cardiac endothelial cell migration and angiogenesis (47). Glucose deficiency can increase the secretion of EXsmiR-126-3p and miR-23a from cardiomyocytes. EXsmiR-126-3p and miR-23a facilitate communication between cardiomyocytes and endothelial cells, thereby promoting endothelial cell proliferation and angiogenesis (48).

### 3.5 Exocrine miRNA and myocardial fibrosis

Changes in the microenvironment and exposure to high glucose can activate inflammatory signals, leading to extracellular collagen matrix deposition in cardiomyocytes and abnormal activation of cardiac fibroblasts. These processes worsen the development of myocardial fibrosis (49). MiRNA plays a significant role in DCM by influencing protein gene expression, thereby affecting the levels of proteins and cytokines involved in myocardial fibrosis (50). Modulating miRNA expression, whether upregulation or downregulation, can regulate downstream target genes, offering a potential effective treatment strategy for improving cardiac function. Myocardial glucose metabolism primarily relies on insulin activation of the insulin signaling pathway IRS-1/2 and downstream PI3K/Akt, which, in turn, stimulates the translocation of glucose transporter 4 (GLUT4) to the cell membrane for glucose uptake and metabolism (51). Notably, it has been observed that patients with type 2 diabetes exhibit an increase in MG53 (mitsugumin53) levels, and this elevated MG53 is associated with the degradation of insulin receptor and IRS-1 proteasome in the central organs of type 2 diabetic mice. This degradation of MG53 further impairs insulin signal transduction. Additionally, the overexpression of MG53 specific to cardiomyocytes inhibits insulin signal transduction and exacerbates myocardial fibrosis (52). In the context of DCM, weakened insulin signal transduction aggravates abnormal glucose metabolism, culminating in myocardial fibrosis and the progression of diabetic cardiomyopathy.

Under conditions of hyperglycemia, advanced glycation end products (AGEs) precipitate within the diabetic heart, leading to the cross-linking of collagen molecules and resulting in myocardial interstitial fibrosis. Several studies have indicated that myocardial interstitial fibrosis is regulated by miR-21, miR-15a/b, miR-133a, miR-29, and miR-200b (53). Liu et al. observed an upregulation of miR-21 expression in cardiac fibroblasts under high glucose conditions, which accelerated collagen synthesis through the c-Jun, N-terminal kinase, and p38 signal pathways. In diabetic patients, myocardial miR-15a/b is downregulated, thereby activating fibrogenic transforming growth factor-β receptor-1 and connective tissue growth factor (54). Feng confirmed that miR-200b mediates the transformation of endothelial cells into mesenchymal cells in diabetic mice, promoting an increase in myocardial interstitial fibrosis in DCM. This suggests that oxidative damage induced by hyperglycemia plays a pivotal role in the pathogenesis. The increase in collagen deposition may be associated with the upregulation of TGF-β and connective tissue growth factor expression, as well as an increase in poly (ADP-ribose polymerase 1) activation under oxidative stress (55). A fundamental analysis has confirmed that exosomes derived from exercising cardiomyocytes decrease the levels of matrix metalloproteinase-9 (MMP-9) by upregulating miR-29b and miR-455. This effect prevents the detrimental consequences of myocardial fibrosis and the uncoupling of cardiomyocytes downstream of MMP-9 (55). The injection of overexpressed cardiomyocyte EXsmiR-146a-5p significantly inhibits the levels of pro-inflammatory cytokines and myocardial fibrosis while restoring cardiac function in a pig model of dilated cardiomyopathy (56). MiR-34a can reduce myocardial fibrosis in DCM by decreasing the production of type I collagen, reducing the viability and migration of fibroblasts, and increasing the apoptosis of fibroblasts through the targeting of Pin-1 signal transduction (57). MiR-223 can activate the NLRP3 inflammasome and lead to cardiac dysfunction in DCM rats. It was found that a miR-223 inhibitor can enhance the morphology and structure of myocardial tissue in DCM model rats, reducing myocardial fibrosis and apoptosis in these rats (58).

Under high-glucose culture conditions, knocking down miR-155 could activate the fibrogenic pathway TGF-β1/Smad2/3, exacerbate collagen I synthesis and cardiomyocyte fibrosis, reduce miR-155 expression, upregulate the transcriptional expression of Tim-3, promote the activation of T lymphocytes towards a Th1 proinflammatory phenotype, and induce the activation of an immuno-inflammatory response in patients with DM. Knocking down LncRNA MALAT1, as demonstrated by Xu (59), may upregulate miR-155, inhibit the activation of the inflammation-fibrosis pathway mediated by Tim-3 and TGF-β1/Smad2/3, and ameliorate myocardial fibrosis in DCM. Bai (60) established a model of type 2 diabetes through intraperitoneal injection of STZ after a high-sugar and high-fat diet, which resulted in decreased weight and increased fasting blood glucose in the DCM group after an initial weight gain. The DCM+ interference miRNA-21 group showed increased body weight and decreased fasting blood glucose after transfection, indicating that miRNA-21 inhibition has a certain effect on body weight and fasting blood glucose in type 2 diabetic rats. The expression of proteins and cytokines in DCM contributes to the development of myocardial fibrosis.

### 3.6 Exocrine miRNA and lipid metabolism

Lipid metabolism is intricately connected to the onset and progression of heart disease. Lipid metabolism plays a crucial role in safeguarding the myocardium by regulating mitochondrial function, apoptosis, or endothelial cell function. This regulation may involve the inhibition of inflammation, the management of oxidative stress, or the control of apoptosis. The intricacy of lipid metabolism is evident in the involvement of numerous enzymes, transporters, and regulatory proteins throughout the process. Multiple signaling pathways work together to provide energy and maintain cell function by regulating the synthesis and breakdown of lipids (61). Nonetheless, research by Xiang et al. has indicated that overexpressing miR-21 in the kidney can disrupt lipid metabolism and contribute to the onset and development of renal fibrosis (62). This effect may be attributed to the dependency of EXs expression on the type and environment of the source cells. Other studies have revealed that miR-320 functions as a small activation RNA in the nucleus at the transcriptional level. It targets the expression of CD36, resulting in increased fatty acid uptake, which leads to cardiac lipotoxicity and cardiac dysfunction (63). MiR-30c can target PGC-1β, reducing the excessive production of reactive oxygen species and myocardial lipid accumulation. This, in turn, alleviates cardiomyocyte apoptosis and cardiac dysfunction in db/db mice (64). Furthermore, miR-122 and miR-33 can regulate fatty acid oxidation by targeting MED13, leading to the accumulation of lipid intermediates like TAG and DAG. This, in turn, affects insulin sensitivity and cardiac function (65).

### 3.7 Exocrine miRNA and mitochondrial dysfunction

Around 90% of ATP in cardiomyocytes is typically generated intracellularly. However, under high glucose conditions, mitochondria shift from glucose to free fatty acids (FFA) oxidation to produce ATP. This process is associated with an increased production of mitochondrial oxidative stress (ROS) and impaired oxidative phosphorylation (21). Mitochondrial reactive oxygen species (ROS) are natural by-products of oxygen metabolism in the electron transport chain complexes I and III. Hyperglycemia and insulin resistance lead to alterations in the transfer of nicotinamide adenine dinucleotide and flavin adenine dinucleotide to the mitochondrial respiratory chain, causing hyperpolarization of the mitochondrial inner membrane. This, in turn, inhibits electron transport in complex III, resulting in excessive ROS production (66). Various miRNAs are involved in the regulation of myocardial energy metabolism. Among them, miR-181c is a miRNA that plays a role in the abnormal regulation of cardiac mitochondrial function, potentially by targeting mitochondrial MT-COX1 mRNA in cardiomyocytes. Upregulation of miR-181c expression can inhibit the expression of mt-COX1 and upregulate the expression level of mt-COX2, leading to a remodeling of mitochondrial respiratory complex IV, ultimately resulting in mitochondrial dysfunction. In ischemic heart failure models, when miR-181c/d is deleted, the heart maintains cardiac function by enhancing the oxidative stress response of mitochondria. This suggests that miR-181c’s interference with mitochondrial function may play a crucial role in myocardial pathophysiology (67). Elizabeth discovered that the expression of the miR-199a/miR-214 cluster is upregulated in human and mouse heart failure models (68). MiR-199a and miR-214 collaborate to regulate peroxisome proliferator-activated receptor delta (PPARδ). PPARδ is a key regulator of cardiac energy metabolism, influencing the transition of cardiac metabolism towards glycolysis during heart failure. Inhibiting the expression of miR-199a and miR-214 can restore the mitochondrial metabolism of FFAs and improve cardiac function.

### 3.8 Exocrine miRNA and autophagy

Mitochondrial autophagy serves to selectively degrade damaged or aging mitochondria. During the development of diabetic cardiomyopathy, mitochondrial autophagy is activated, and its impact can be dual in nature. The enhancement of mitochondrial autophagy has the potential to mitigate mitochondrial dysfunction, decrease lipid accumulation, and prevent cardiac dysfunction. However, excessive autophagy may exacerbate diabetic cardiomyopathy (69). Research has confirmed that exosomal miRNAs can activate autophagy and delay apoptosis in various cells by regulating different signaling pathways. Exosomal miRNAs related to autophagy include miR-20a-5p, miR-130a, miR-126, and miR-21. Under high glucose conditions, miR-20a-5p inhibits the overexpression of phosphatase and tensin homolog (PTEN) genes and Atg7 in endothelial progenitor cells (EPCs), thereby activating the Akt/mTOR pathway to inhibit excessive autophagy in EPCs and promote angiogenesis (70). Overexpression of miR-30c can inhibit the induction of BECN1 (a tumor suppressor gene antibody) and subsequent autophagy in the myocardium of diabetic mice, resulting in improved cardiac structure and function (71). The expression of miR-34a is upregulated in high glucose-induced cardiomyocytes and the hearts of diabetic mice, where it acts to inhibit autophagy in cardiomyocytes and heart tissues (72).

## 4 Diagnostic potential of exocrine miRNA in DCM

It has been discovered that exosomal miRNA (EXsmiRNA) can serve as a non-invasive biomarker for detecting DCM. In comparison to mice on a normal diet, insulin-resistant mice induced by a high-fat diet showed significantly elevated levels of circulating miR-1 and miR-133a, indicating greater myocardial steatosis. Similarly, patients with T2DM and myocardial steatosis had higher levels of miR-1 and miR-133a in their cardiomyocytes when compared to healthy individuals. Furthermore, lipid-loaded HL-1 cardiomyocytes also released increased amounts of EXsmiR-1 and miR-133a (73).

In contrast, plasma miR-21 levels were significantly lower in the DCM group of T2DM patients when compared to those with T2DM but without DCM. The diagnostic efficiency of miR-21 (AUC = 0.899) was notably higher than HbA1C and other parameters (74). Additionally, EXsmiRNA, specifically miR-1, miR-133a, and miR-21, may serve as potential biomarkers for diabetic myocardial injury. EXsmiR-21 derived from cardiac progenitor cells can protect cardiomyocytes from apoptosis induced by oxidative stress by downregulating the expression of programmed cell death factor 4 (pdd4) (75).

Both miR-19b and miR-181b are associated with the decline in cardiac function in diabetic mice. MiR-19b is upregulated in the myocardium of diabetic mice, while miR-181b plays a central role in vascular inflammation, inhibiting the activation of the NF-κB signaling pathway in endothelial cells (76). Furthermore, miR-181b is a key player in vascular remodeling, activating the TGF-β/pSmadD2/3 pathway and thus may serve as a potential biomarker for diabetic cardiomyopathy (77). The serum levels of miR-1 and miR-133a in cardiomyocytes serve as predictors of myocardial steatosis in well-controlled short-term type 2 diabetic patients without complications (73).

## 5 Treatment of diabetic cardiomyopathy with exocrine miRNA

### 5.1 Exocrine bodies derived from stem cells

The impact of exosomes derived from stem cells on cardiomyocyte repair and the protection of cardiac function and structure has been increasingly recognized. Exosomal miR-146a from bone marrow mesenchymal stem cells (bmMSC) reversed the decline in islet β-cell function and insulin secretion by regulating the NUMB/β-catenin signaling pathway (78). Exosomes released by human embryonic stem cell (ESC)-derived mesenchymal stem cells (ESC-MSCs) were found to protect myocardium from injury both *in vitro* and *in vivo*, potentially due to the increased levels of cardioprotective Akt and GSK3 α/β, as reported in a study by LaiRC (79, 80). Khans’s research indicates that exosomes from embryonic stem cells can transfer their contents, such as miR-294, to adjacent target cells, which results in improved myocardial necrosis in ischemic cardiomyopathy. This leads to increased new microangiogenesis, myocyte proliferation, and enhanced survival following myocardial infarction (81).

Furthermore, the high levels of miRNA-218-5p and miRNA-363-3p in endothelial progenitor cell (EPC)-derived exosomes promote fibroblast proliferation and the transformation of endothelial cells to a mesenchymal state by up-regulating p53 and down-regulating JMY, effectively inhibiting myocardial fibrosis. Administering miRNA-218-5p or miRNA-363-3p analogs in a mouse acute myocardial infarction (AMI) model reduces myocardial infarction size and enhances myocardial repair post-infarction (82). Luther et al. confirmed that MSC exosomes enriched with miRNA-21a-5p can inhibit the expression of pro-apoptotic genes like PDCD4, PTEN, Peli1, and FasL in cardiomyocytes, leading to anti-apoptotic effects and reduced myocardial infarction volume in mice (83).

### 5.2 Exocrine body derived from cardiac cells

Several exosomes derived from cardiac cells can also enhance myocardial function and ameliorate the myocardial tissue microenvironment. Exosomes from exercised cardiomyocytes reduce the levels of matrix metalloproteinase-9 (MMP-9) by up-regulating miR-29b and miR-455, thereby mitigating the adverse effects of myocardial fibrosis and the downstream uncoupling of cardiomyocytes caused by MMP-9 (55). Exosomes derived from cardiosphere-derived cells promote angiogenesis and cardiac regeneration in scarred and infarcted hearts through the activation of miRNA-146a, offering protection against oxidative stress (84). Exosomes produced by cardiac progenitor cells (CPC) under anoxic conditions enhance angiogenesis potential and, furthermore, reduce the expression of fibrogenic genes in rat fibroblasts stimulated by TGF-β (85).

### 5.3 Exocrine as carriers

Research on exosomes and miRNA has advanced gene-targeted therapy for cardiovascular diseases. Gene therapy’s biomolecules are inherently unstable *in vivo* and are susceptible to destruction or degradation by nucleases before they can reach their target cells, making it challenging for them to reach the target sites and be effective. Therefore, the selection of efficient and safe gene delivery vectors is crucial. In comparison to commonly used viral carriers, exosomes, as drug delivery carriers, offer strong membrane stability, targeting capabilities, and high efficiency. Wei et al. harnessed the targeting function of exosomes to deliver miRNA-181a into the hearts of mice, leading to increased levels of regulatory T cells and anti-inflammatory factors, ultimately reducing reperfusion injury after myocardial infarction (86). Chachques et al. introduced macrophages and exosome-rich MSCs onto elastic scaffolds to enhance myocardial repair and regeneration by modulating miRNA-124 expression and reducing miRNA-125 expression (87).

### 5.4 Natural medicine

In recent years, research related to treating diabetic cardiomyopathy with natural products through the regulation of exosomal miRNA has been on the rise, becoming a burgeoning research focus. Berberine (BBR) is a natural compound found in various Chinese herbal medicines and possesses a range of biological activities. Numerous studies have confirmed the anti-inflammatory, antipsychotic, and significant neuroprotective effects of berberine (88). BBR targets Gsdmd by upregulating miR-18a-3p, thereby mitigating pyroptosis in H9C2 cells treated with high glucose. Furthermore, BBR alleviates diabetic cardiomyopathy in a rat model by inhibiting miR-18a-3p-mediated Gsdmd activation (89). Dihydromyricetin is a natural flavonoid with notable antifibrotic effects, primarily through its antioxidative, anti-inflammatory, collagen production inhibition, and autophagy regulation properties (90). Dihydromyricetin improves diabetic cardiomyopathy by reducing miR-34a expression and promoting cardiomyocyte autophagy (72).

## 6 Discussion and conclusion

The pathogenesis of diabetic cardiomyopathy primarily involves abnormal energy metabolism and microangiopathy. Several exosomal miRNAs are closely associated with the onset and progression of DCM. Differentially expressed miRNAs and protein molecules in exosomes can serve as early predictive or diagnostic biomarkers for AMI. Moreover, leveraging the unique characteristics of exosomes, exosomes from various sources or exosomes as drug carriers hold promise for enhancing cardiac function and clinical outcomes, offering potential prospects for clinical treatment. This review provides a comprehensive summary of the current understanding of the roles of exosomes and their miRNAs in DCM. Several studies have demonstrated the potential therapeutic efficacy and biomarker capabilities of exosomal miRNAs.

Our study comprehensively outlines the pathogenic mechanisms underlying diabetic cardiomyopathy, illuminating the intricate interplay of oxidative stress, inflammation, and fibrosis in the progression of this disease. We highlight the groundbreaking role of exosomes in intercellular communication within the cardiovascular system, emphasizing a significant advancement in our understanding. We discovered that exosomes and exosomal microRNAs play a crucial role as essential carriers in facilitating the transfer of information between cells. This intricate web of intercellular communication not only shapes cell behavior but also holds substantial potential for the diagnosis and treatment of diabetic cardiomyopathy. Special attention is given to specific microRNAs, such as miR-30c, miR-133a, and miR-146, serving as key focal points of our research. By identifying these molecular participants, our paper contributes to a more profound comprehension of the molecular mechanisms at the core of diabetic cardiomyopathy. The recognition of exosomal microRNAs as potential biomarkers for early diagnosis and prognosis adds a layer of clinical relevance to our study, presenting a promising pathway for the implementation of precision medicine in the treatment of diabetic cardiomyopathy. Moreover, the article delves into the challenges and opportunities linked to gene-targeted therapies for cardiovascular diseases, offering a thorough exploration of exosomal microRNAs as potential therapeutic tools. Acknowledging the inherent instability of biological molecules in the body and the demand for more effective delivery systems, our article provides a realistic assessment of the current prospects for gene-targeted therapies, while also outlining future research directions aimed at overcoming these challenges. This contributes to the ongoing efforts to continually enhance drug development and deepen our understanding of the mechanisms underlying diabetic cardiomyopathy. However, it is important to note that the research on exosomal miRNAs in DCM is still in its early stages, and the specific underlying mechanisms warrant further exploration. Future investigations are expected to delve deeper into the realm of exosomal miRNAs in DCM, aiming to deliver more effective treatments for mending diabetic myocardial injuries.

## Author contributions

SG: Writing – original draft. YD: Writing – original draft. CY: Writing – original draft. TY: Writing – original draft. HC: Writing – review & editing.
